# HPV prevalence and genotype distribution in a population-based split-sample study of well-screened women using CLART HPV2 Human Papillomavirus genotype microarray system

**DOI:** 10.1186/1471-2334-14-413

**Published:** 2014-07-26

**Authors:** Jesper Bonde, Matejka Rebolj, Ditte Møller Ejegod, Sarah Preisler, Elsebeth Lynge, Carsten Rygaard

**Affiliations:** Department of Pathology, Copenhagen University Hospital, Hvidovre, Kettegård Allé 30, 2650 Hvidovre, Denmark; Clinical Research Center, Copenhagen University Hospital, Hvidovre, Kettegård Allé 30, 2650 Hvidovre, Denmark; Department of Public Health, University of Copenhagen, Øster Farimagsgade 5, 1014 Copenhagen K, Denmark

**Keywords:** Human papillomavirus, Cervical cancer, Genotyping, Screening, Assay, CLART, HC2, Vaccine monitoring

## Abstract

**Background:**

Human papillomavirus (HPV) genotyping assays are becoming increasingly attractive for use in mass screening, as they offer a possibility to integrate HPV screening with HPV vaccine monitoring, thereby generating a synergy between the two main modes of cervical cancer prevention. The Genomica CLART HPV2 assay is a semi-automated PCR-based microarray assay detecting 35 high-risk and low-risk HPV genotypes. However, few reports have described this assay in cervical screening.

An aim of the present study, Horizon, was to assess the prevalence of high-risk HPV infections in Copenhagen, Denmark, an area with a high background risk of cervical cancer where women aged 23-65 years are targeted for organized screening.

**Methods:**

Material from 5,068 SurePath samples of women participating in routine screening and clinical follow-up of cervical abnormalities was tested using liquid based cytology, CLART HPV2 and Hybrid Capture 2 (HC2).

**Results:**

At least one of the 35 defined genotypes was detected by CLART in 1,896 (37%) samples. The most frequent high-risk genotypes were HPV 16 (7%), HPV 52 (5%), and HPV 31 (4%). The most frequent low-risk genotypes were HPV 53 (5%), HPV 61 (4%), and HPV 66 (3%). Among 4,793 women targeted by the screening program (23-65 years), 1,166 (24%) tested positive for one or more of the 13 high-risk genotypes. This proportion decreased from 40% at age 23-29 years to 10% at age 60-65 years. On HC2, 1,035 (20%) samples were positive for any high-risk and thus CLART showed a higher analytical sensitivity for 13 high-risk HPV genotypes than HC2, and this was found in all age-groups and in women normal cytology.

**Conclusions:**

CLART performed well with a positive reproducibility for high-risk genotypes of 86%, and a negative reproducibility of 97%. This report furthermore updates the genotype distribution in Denmark prior to the inclusion of the HPV-vaccinated cohorts into the screening program, and as such represents a valuable baseline for future vaccine impact assessment.

**Electronic supplementary material:**

The online version of this article (doi:10.1186/1471-2334-14-413) contains supplementary material, which is available to authorized users.

## Background

Human Papillomavirus (HPV) infection is a necessary cause of cervical cancer [1]. At present, 13 genotypes (16, 18, 31, 33, 35, 39, 45, 51, 52, 56, 58, 59, 68) are considered high-risk or probably high-risk [2]. Molecular assays detecting DNA from these HPV genotypes, in particular the commercially available Hybrid Capture 2 (HC2; Qiagen, Hilden, Germany) and in-house polymerase chain reaction (PCR) using GP5+/6+ primers, have been extensively studied in randomized controlled trials. In these trials, a high sensitivity for detection of high-grade cervical intraepithelial neoplasia (CIN) was demonstrated for HPV testing compared to cytology [3, 4]. This makes molecular HPV testing attractive for future cervical screening. Since 2006, vaccination against genotypes 16 and 18 has been available, primarily for younger birth cohorts. In Denmark, for example, HPV vaccination has been recommended in women under 26 years of age. The lower background risk of cervical cancer expected in vaccinated women is expected to reduce the positive predictive value of cytology [5], reinforcing the case for a shift towards HPV-based screening.

Several HPV assays are now commercially available. The most frequently used assay, the Hybrid Capture 2 (HC2), detects a combination of 13 genotypes without individual genotyping. However, inclusion of HPV16 and 18 in the present vaccines, and a higher number of genotypes in next generation vaccines call for more detailed monitoring of genotypes. For this purpose, HPV assays with genotype resolution will be necessary. Commercially available full-genotyping assays, e.g. Linear Array (Roche, Rotkreuz, Switzerland) detecting 37 high- and low-risk genotypes, or INNO-LiPA (Innogenetics, Gent, Belgium) detecting 28 genotypes, have been extensively used in research. However, their results are most often read manually, and the tests are therefore prone to inter-observer variability making quality assurance for use in routine screening a challenge.

The CLART HPV2 (Genomica, Madrid, Spain) assay offers an alternative for genotype specific screening covering 35 individual HPV genotypes, including all 13 high-risk or probably high-risk genotypes. A set of primers to amplify a fragment of the human CFTR gene (892 bp) is included as a DNA control for sample adequacy, while an additional set of primers to amplify a spiked plasmid (1202 bp) is included as a PCR process control. The resulting visualized microarray is analysed on a computer guided reader with automated reading software. This reduces inter-observer variation in reporting the results, and is useful for high-throughput settings. Furthermore, CLART is a versatile high-throughput assay that can be applied to several sample types for HPV diagnostics, including formalin fixed paraffin embedded specimens [6–8]. Versatility and speed are becoming increasingly crucial as laboratories, in supporting the clinical diagnostic processes, undertake HPV testing on a wide variety of sample types from the same patient.

Earlier studies reported mixed results when the detection of HPV and cervical lesions by CLART was compared to that of HC2 [8–13]. All studies were undertaken using samples stored in PreservCyt, and experts have called for evaluation of HPV assays using other storage media [14], e.g. SurePath. Here we present data from the Horizon study comparing the outcomes of the CLART and HC2 assays. The Horizon study is a split-sample, population-based study of well-screened women with a high background risk of cervical cancer [15, 16], using samples stored in SurePath. Moreover, the data presented here will be the last population-based Danish data on HPV prevalence in virtually unvaccinated women, and as such form a valuable baseline for future assessment of vaccine impact.

## Methods

### Setting

The Department of Pathology of Copenhagen University Hospital in Hvidovre is the largest cervical screening laboratory in Denmark. While the study was on-going, it annually evaluated 66,000 cervical SurePath samples from women living in the municipalities of Copenhagen and Frederiksberg (denoted as Copenhagen in the remainder of the manuscript). The laboratory handles all cervical cytology regardless of the reason for sample-taking. Since the 1960’s, Copenhagen has been covered by an organized cervical screening program. At present, women aged 23-49 years are targeted for screening every three years and women aged 50-65 years every five years. In 2012, 75% of women had been screened at least once within the recommended interval [17].

### Sample collection

The Horizon study was nested into routine laboratory practice. Upon arrival in the laboratory, SurePath samples were sequentially arranged into racks of 48. Samples from the first one to four racks were collected for the study, equally from Monday to Friday, between 10 June and 25 August 2011. This collection method mimicked a cohort of unselected consecutive liquid-based cytology (LBC) samples, assuming that the time the sample arrived in the laboratory was not associated with the woman’s characteristics. Residual material was collected after completion of routine LBC which included HC2 triage of women aged ≥30 years with atypical squamous cells of undetermined significance (ASCUS). The volume of the collected residual material was approximately 2 ml per sample, and it was diluted with 2 ml of SurePath (dilution factor approximately 1:1) to obtain enough volume for additional testing reported elsewhere [18–20]. All testing was done in the same laboratory. In agreement with the manufacturers, taking into account the additional workload in this routine laboratory and the cost, the target number of samples was 5,000.

### Cytology

Cytological evaluation of SurePath samples was undertaken by cytoscreeners, though in collaboration with a pathologist in case of abnormal findings. Reading was assisted by FocalPoint GS Imaging System (BD Diagnostics, Burlington, NC, USA). The outcomes were reported using the Bethesda 2001 system. They were classified as negative for intraepithelial lesion or malignancy, ASCUS, low-grade squamous intraepithelial lesions (LSIL), or high-grade squamous intraepithelial lesions or worse (≥HSIL) including atypical squamous cells - cannot exclude HSIL (ASC-H), atypical glandular cells (AGC), adenocarcinoma in situ (AIS), and squamous cell carcinoma. Cytoscreeners and pathologists were blinded to the outcomes of HPV testing.

### CLART HPV DNA testing

CLART HPV2 (Genomica, Madrid, Spain) is a low-density microarray assay based on PCR amplification of genotype specific HPV L1 fragments from 35 individual HPV genotypes (6, 11, 16, 18, 26, 31, 33, 35, 39, 40, 42, 43, 44, 45, 51, 52, 53, 54, 56, 58, 59, 61, 62, 66, 68, 70, 71, 72, 73, 81, 82, 83, 84, 85 and 89), with analytical sensitivity calibrated against known copies of cloned plasmids.

One ml of the diluted SurePath sample was spun down (5 min, 14,000 revolutions per minute), with the supernatant removed and cell pellet resuspended in a mix of 180 μl phosphate buffered saline (10x conc. pH 7.4, pharmacy product) and 20 μl Proteinase K (recombinant, PCR Grade, Roche Diagnostics). Samples were then vortexed and incubated for one hour at 56°C and one hour at 90°C. HPV DNA was purified using the MagNa Pure LC 96 and MagNA Pure LC 32 instruments with the MagNA Pure LC Total Nucleic Acid Isolation Kit (all Roche Diagnostics, Rotkreutz, CH). Five μl of purified DNA were used for the PCR amplification. During amplification the PCR products were labeled with biotin. Prior to visualization, the PCR products were denatured at 95°C for 10 minutes. Visualization was performed on the CLART microarray, using 10 μl of the denatured PCR products. Hybridization between the amplicons and their specific probes on CLART results in formation of an insoluble precipitate of peroxidase when adding a Streptavidin conjugate that bind to the biotin-labeled PCR products. Precipitate is analyzed on the Clinical Array Reader (Genomica, Madrid, Spain). All samples returning an invalid outcome were retested, and the second result was considered definitive.

Samples for evaluation of CLART’s intra-laboratory reproducibility were collected, in part, within the Horizon study. To obtain sufficient numbers of positive and negative samples for the analysis, Horizon samples were supplemented with other routine screening samples. Extracted DNA was used for this purpose, in order to minimize any influence of the MagNA Pure step.

### Hybrid capture 2 HPV DNA testing

HC2 is a hybridization assay detecting a combination of 13 high-risk genotypes, without an internal control for sufficiency of sample material. SurePath samples were either pretreated manually with DNA denatured prior to testing according to the manufacturer’s protocol, or DNA was isolated and purified using the DSP AXpH DNA kit on the QIASymphony SP platform (Qiagen, Hilden, Germany). Testing of these samples was performed on automated Rapid Capture^®^ System (Qiagen, Gaithersburg, MD) using scripts depending on pretreatment. A small number of samples from women with ASCUS aged ≥30 years, in whom HPV triage was undertaken routinely, were denatured and tested manually.

### Processing of samples and assay instrumentation

The study protocol and assay testing protocols were agreed upon with all manufacturers prior to the study. All instrumentation and software were used as supplied and maintained by the manufacturers.

### Screening history

Women’s screening history from 1 January 2000 onwards was retrieved from the Danish Pathology Data Bank [21], where the reason for taking the sample has not been systematically registered. Horizon samples with an earlier diagnosis of cervical cancer, a CIN diagnosis up to three years earlier, ASCUS in the previous 15 months, or with more severe cytological abnormalities or a positive HPV test in the past 12 months were considered follow-up samples. Horizon samples with no recent abnormality were considered primary samples. The latter included screening samples and a small proportion of samples taken for indication.

### Statistical analyses

CLART was assigned a positive result if it detected at least one of the 13 high-risk HPV genotypes that are included in HC2; HC2 if relative light unit to cut-off (rlu/co) value was ≥1, and cytology when ≥ ASCUS. Differences in the distributions of age, screening history, cytology and HC2 outcomes between included and excluded samples were tested with the *χ*^2^ test. Trends in HPV positivity by age were tested with the Mantel-Haenszel *χ*^2^ test for trend.

### Ethical considerations

Horizon was designed as a quality development study, utilizing only residual material that would otherwise have been discarded. According to Danish regulations of biomedical research, published on 5 May 2011 in the Guidelines about Notification etc. of a Biomedical Research Project to the Committee System on Biomedical Research Ethics No. 9154 section 2.5, quality development studies do not require ethical approval. The study was notified to the Danish Data Protection Agency in concordance with the current guidelines (The Act on Processing of Personal Data, Act No. 429 of 31th May, 2000).

## Results

### Samples

Of the 12,138 consecutive samples received in the laboratory during the collection period, 6,258 (52%) were selected for the study. All selected samples were tested with HC2 except 23 (0.4%), and with LBC. In total, 1,190 (19%) samples were not tested with CLART owing to insufficient residual volume (n = 1,165) or to human error (n = 25), and were excluded. Excluded samples were not significantly different from the remaining 5,068 samples in terms of age (P = 0.09), cytology (P = 0.55), and HC2 outcome (P = 0.31). There was a small statistically significant difference in the women’s screening history, with 13% of the included and 10% of the excluded samples being follow-up samples (P = 0.01). Five thousand and nine women contributed one sample (99% of all samples), 28 contributed two, and one woman three samples. The mean age of the women was 37.3 years (SD = 12.3, range: 16-89). The mean number of days between the receipt of the sample in the laboratory and storing was 2 (range: 1-5).

### Genotype frequency, and single versus multiple infections

**Figure 1:**
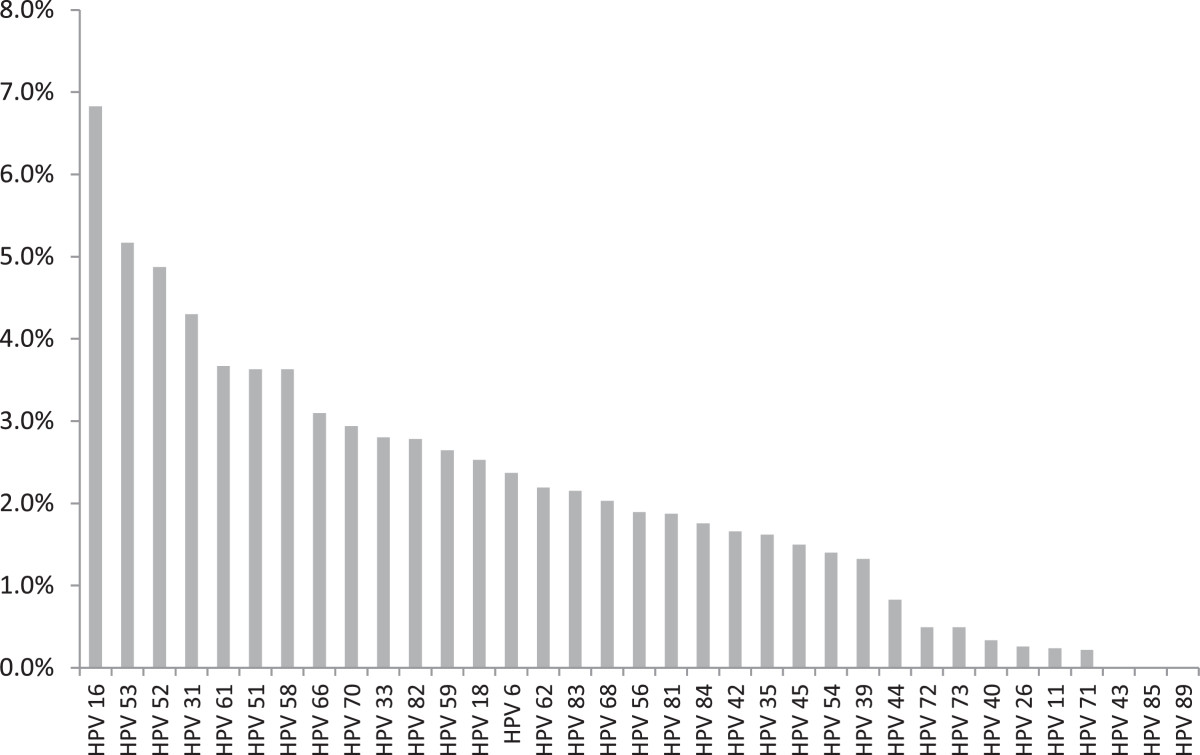
**Genotype distribution on 5,068 unselected samples tested on CLART.**

At least one of 35 genotypes was detected by CLART in 1,896 (37%) samples (Table 1). The most frequent high-risk genotypes were HPV 16 (n = 346, 7%), HPV 52 (n = 247, 5%), and HPV 31 (n = 218, 4%, Figure 1). Of all 1,274 (25%) samples with high-risk genotypes, 496 (39%) were single infections. The most frequent low-risk genotypes were HPV 53 (n = 262, 5%), HPV 61 (n = 186, 4%), and HPV 66 (n = 157, 3%). From 1,207 samples with infections with low-risk genotypes, 492 (41%) were single infections, and 622 (52%) were purely low risk infections with no detected HR-genotypes in the sample.

**Table 1:** **Distribution of infections in the 5,068 samples, by HPV genotype as detected by CLART**

	Infections		
HPV genotypes	Single (%)	Multiple (%)	Total (%)
**High-risk** ^**a**^			
**16**	120 (35%)	226 (65%)	346 (100%)
**18**	31 (24%)	97 (76%)	128 (100%)
**31**	44 (20%)	174 (80%)	218 (100%)
**33**	35 (25%)	107 (75%)	142 (100%)
**35**	18 (22%)	64 (78%)	82 (100%)
**39**	17 (25%)	50 (75%)	67 (100%)
**45**	14 (18%)	62 (82%)	76 (100%)
**51**	41 (22%)	143 (78%)	184 (100%)
**52**	79 (32%)	168 (68%)	247 (100%)
**56**	18 (19%)	78 (81%)	96 (100%)
**58**	45 (24%)	139 (76%)	184 (100%)
**59**	31 (23%)	103 (77%)	134 (100%)
**68**	3 (3%)	100 (97%)	103 (100%)
**≥1 high-risk genotype**	496 (39%)	778 (61%)	1,274 (100%)
**≥1 high-risk genotype, no low-risk genotype**	496 (72%)	193 (28%)	689 (100%)
**Low-risk** ^**a**^			
**6**	34 (28%)	86 (72%)	120 (100%)
**11**	4 (33%)	8 (67%)	12 (100%)
**26**	2 (15%)	11 (85%)	13 (100%)
**40**	1 (6%)	16 (94%)	17 (100%)
**42**	20 (24%)	64 (76%)	84 (100%)
**43**	0	0	0
**44**	5 (12%)	37 (88%)	42 (100%)
**53**	69 (26%)	193 (74%)	262 (100%)
**54**	20 (28%)	51 (72%)	71 (100%)
**61**	58 (31%)	128 (69%)	186 (100%)
**62**	36 (32%)	75 (68%)	111 (100%)
**66**	52 (33%)	105 (67%)	157 (100%)
**70**	55 (37%)	94 (63%)	149 (100%)
**71**	4 (36%)	7 (64%)	11 (100%)
**72**	10 (40%)	15 (60%)	25 (100%)
**73**	5 (20%)	20 (80%)	25 (100%)
**81**	27 (28%)	68 (72%)	95 (100%)
**82**	20 (14%)	121 (86%)	141 (100%)
**83**	45 (41%)	64 (59%)	109 (100%)
**84**	25 (28%)	64 (72%)	89 (100%)
**85**	0	0	0
**89**	0	0	0
**≥1 low-risk genotype**	492 (41%)	715 (59%)	1,207 (100%)
**≥1 low-risk genotype, no high-risk genotype**	492 (79%)	130 (21%)	622 (100%)
**≥1 high-risk or low-risk genotype**	988 (52%)	908 (48%)	1,896 (100%)

*Abbreviations:* *HPV* Human Papillomavirus.

^a^According to the classification reported by IARC [2].

Among 4,793 women targeted by the screening program (23-65 years), 1,166 (24%) tested positive for high-risk genotypes (Table 2). This proportion decreased from 40% at age 23-29 years to 10% at age 60-65 years (P < 0.0001). Infections with only low-risk genotypes showed a small but significant decrease by age (P = 0.03). In women aged 16-22 and >65 years, 61% and 8%, respectively, tested positive on high-risk genotypes. These women are not routinely invited for screening, but may have presented for medical conditions, sought screening themselves, or were tested for follow-up of an earlier abnormality.

**Table 2:** **CLART results for 5,068 samples, by age, screening history, cytology, and HC2 results**

	Outcomes on CLART, N (%)				
	Any high-risk HPV genotype (%)^a^	Negative on high-risk HPV genotypes (%)^b^	Only low-risk HPV genotypes (%)^a^	Invalid (%)	Total (%)
**Total**	1,274 (25.1%)	3,782 (74.6%)	622 (12.3%)	12 (0.2%)	5,068 (100%)
**Age (years)**					
16-22	99 (61.1%)	63 (38.9%)	20 (12.3%)	0 (0.0%)	162 (100%)
23-29	608 (39.6%)	926 (60.3%)	216 (14.1%)	1 (0.1%)	1,535 (100%)
30-39	345 (22.6%)	1,176 (77.1%)	171 (11.2%)	4 (0.3%)	1,525 (100%)
40-49	135 (13.6%)	851 (85.8%)	119 (12.0%)	6 (0.6%)	992 (100%)
50-59	54 (10.7%)	452 (89.2%)	55 (10.8%)	1 (0.2%)	507 (100%)
60-65	24 (10.3%)	210 (89.7%)	25 (10.7%)	0 (0.0%)	234 (100%)
>65	9 (8.0%)	104 (92.0%)	16 (14.2%)	0 (0.0%)	113 (100%)
**Screening history**					
Primary sample^c^	1,024 (23.2%)	3,375 (76.5%)	528 (12.0%)	11 (0.2%)	4,410 (100%)
Follow-up sample	250 (38.0%)	407 (61.9%)	94 (14.3%)	1 (0.2%)	658 (100%)
**Cytology**					
Normal	1,001 (21.4%)	3,658 (78.3%)	561 (12.0%)	12 (0.3%)	4,671 (100%)
ASCUS	72 (58.5%)	51 (41.5%)	18 (14.6%)	0 (0.0%)	123 (100%)
LSIL	103 (72.5%)	39 (27.5%)	35 (24.6%)	0 (0.0%)	142 (100%)
≥HSIL	97 (91.5%)	9 (8.5%)	5 (4.7%)	0 (0.0%)	106 (100%)
≥ASCUS	272 (73.3%)	99 (26.7%)	58 (15.6%)	0 (0.0%)	371 (100%)
Inadequate	1 (3.8%)	25 (96.2%)	3 (11.5%)	0 (0.0%)	26 (100%)
**HC2** ^**d**^					
Positive	874 (84.4%)	160 (15.5%)	111 (10.7%)	1 (0.1%)	1,035 (100%)
Negative	399 (9.9%)	3,619 (89.8%)	510 (12.7%)	11 (0.3%)	4,029 (100%)

*Abbreviations:* *ASCUS* atypical squamous cells of undetermined significance, *HC2* Hybrid Capture 2 assay, *HPV* Human Papillomavirus, *≥HSIL* high-grade intraepithelial lesions or worse, *LSIL* low-grade squamous intraepithelial lesions.

^a^Thirteen high-risk and probably high-risk genotypes, according to the classification reported by IARC [2].

^b^None of the 13 high-risk or probably high-risk genotypes detected.

^c^Predominantly screening samples, including a small proportion of samples taken for indication.

^d^Four samples were not tested with HC2.

### Concurrent cytology

Cytological abnormalities were detected in 371 (7%) of samples. The proportion of CLART positive samples increased by cytological grade; 1,001 (21%) samples with normal cytology were positive on CLART; 72 (59%) with ASCUS; 103 (73%) with LSIL; and 97 (92%) with ≥ HSIL (P < 0.0001). The small proportion of samples with only low-risk genotypes showed no trend by cytological grade (P = 0.28). Among women aged 23-65 years, HPV 16 was found in 235 (5%), and HPV 18 in 88 (2%) of 4,435 samples with normal cytology (Table 3). Furthermore, HPV 16 was found in 16 (14%) of ASCUS; 27 (21%) of LSIL; and 37 (38%) of ≥ HSIL samples; whereas HPV 18 was found in 7 (6%) of ASCUS; 7 (5%) of LSIL; and 15 (16%) of ≥ HSIL samples. Among cytology normal samples at this age, at least one high-risk genotype was detected in 923 (21%), whereas this was the case in 242 (72%) of cytology abnormal samples. Multiple infections with high-risk genotypes were more frequent than single infections regardless of the grade of cytological abnormality. Infections with only low-risk genotypes were detected in 530 (12%) cytology normal, and in 53 (16%) cytology abnormal samples.

**Table 3:** **Prevalence of high-risk and low-risk HPV genotypes as detected by the CLART assay in women aged 23–65 years, by cytology**

	Cytology result				
HPV genotype	Normal (N = 4,435)	ASCUS (N = 110)	LSIL (N = 129)	≥HSIL (N = 96)	≥ASCUS (N = 335)
**HPV 16**	235 (5.3%)	16 (14.5%)	27 (20.9%)	37 (38.5%)	80 (23.9%)
**HPV 18**	88 (2.0%)	7 (6.4%)	7 (5.4%)	15 (15.6%)	29 (8.7%)
**HPV 31**	157 (3.5%)	8 (7.3%)	20 (15.5%)	16 (16.7%)	44 (13.1%)
**HPV 33**	98 (2.2%)	6 (5.5%)	13 (10.1%)	8 (8.3%)	27 (8.1%)
**HPV 35**	52 (1.2%)	8 (7.3%)	6 (4.7%)	3 (3.1%)	17 (5.1%)
**HPV 39**	38 (0.9%)	5 (4.5%)	15 (11.6%)	2 (2.1%)	22 (6.6%)
**HPV 45**	43 (1.0%)	8 (7.3%)	9 (7.0%)	9 (9.4%)	26 (7.8%)
**HPV 51**	139 (3.1%)	8 (7.3%)	10 (7.8%)	6 (6.3%)	24 (7.2%)
**HPV 52**	171 (3.9%)	14 (12.7%)	12 (9.3%)	24 (25.0%)	50 (14.9%)
**HPV 56**	52 (1.2%)	6 (5.5%)	24 (18.6%)	1 (1.0%)	31 (9.3%)
**HPV 58**	133 (3.0%)	12 (10.9%)	12 (9.3%)	10 (10.4%)	34 (10.1%)
**HPV 59**	90 (2.0%)	8 (7.3%)	13 (10.1%)	5 (5.2%)	26 (7.8%)
**HPV 66**	115 (2.6%)	5 (4.5%)	22 (17.1%)	2 (2.1%)	29 (8.7%)
**HPV 68**	68 (1.5%)	4 (3.6%)	14 (10.9%)	8 (8.3%)	26 (7.8%)
**≥1 high-risk genotype** ^**a**^	923 (20.8%)	61 (55.5%)	93 (72.1%)	88 (91.7%)	242 (72.2%)
**Single infection with a high-risk genotype** ^**a**^	388 (8.7%)	22 (20.0%)	20 (15.5%)	37 (38.5%)	79 (23.6%)
**Multiple infection including high-risk genotype(s)** ^**a**^	535 (12.1%)	39 (35.5%)	73 (56.6%)	51 (53.1%)	163 (48.7%)
**≥1 low-risk genotype** ^**a**^	943 (21.3%)	40 (36.4%)	88 (68.2%)	34 (35.4%)	162 (48.4%)
**≥1 low-risk genotype, no high-risk genotype** ^**a**^	530 (12.0%)	17 (15.5%)	32 (24.8%)	4 (4.2%)	53 (15.8%)
**No high- or low-risk genotype**	2,970 (67.0%)	32 (29.1%)	4 (3.1%)	4 (4.2%)	40 (11.9%)

*Abbreviations:* *ASCUS* atypical squamous cells of undetermined significance, *HC2* Hybrid Capture 2 assay, *HPV* Human Papillomavirus, *≥HSIL* high-grade intraepithelial lesions or worse, *LSIL* low-grade squamous intraepithelial lesions.

^a^Categorization of HPV genotypes into high-risk and low-risk groups followed IARC’s classification [2], according to which genotype 66 is considered “possibly carcinogenic” (low-risk).

### CLART versus HC2

CLART could be compared to HC2 on 5,064 samples, as four samples were not tested with HC2. Here, 1,035 (20%) samples were positive on HC2, HC2 being positive in 874 (69%) CLART-positive samples, and in 160 (4%) CLART-negative samples. The majority, 111 (69%), of CLART-negative/HC2 positive samples tested positive on CLART low-risk genotypes, indicating some HC2 cross-reactivity to low-risk HPV genotypes. HC2 showed slightly lower prevalence of high-risk HPV infections than CLART in all age groups; the prevalence decreased from 57% (92/162) at age 16-22 years, to 33% (507/1,534) at age 23-29 years, 17% (266/1,525) at age 30-39 years, 11% (112/991) at age 40-49 years, 7% (37/506) at age 50–59 years, 6% (14/234) at age 60-65 years, and 6% (7/112) at age >65 years. Compared to CLART, HC2 tested positive in fewer samples with normal cytology, 16% (728/4,667), but in more samples with low-grade abnormal cytology, 64% (79/123) in ASCUS, and 90% (128/142) in LSIL. Both assays tested positive on 92% (97/106) of samples diagnosed as ≥ HSIL; on 94 of these 97 samples both CLART and HC2 returned a positive result, whereas three samples tested positive on CLART and negative on HC2, and additional three samples tested negative on CLART and positive on HC2.

### Outcome in primary screening samples

**Figure 2:**
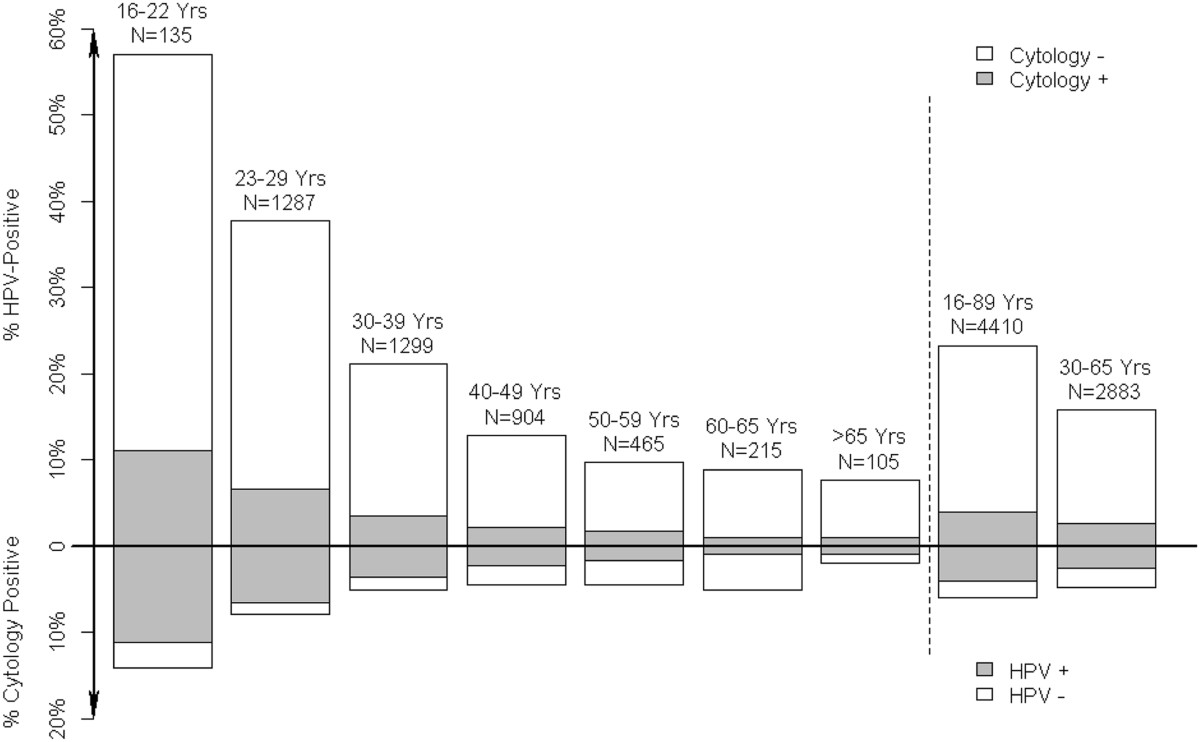
**Proportions of the 4,410 screening samples testing positive on CLART, and with abnormal cytology, by age.**

When only the 4,410 primary screening samples without a recent abnormality were considered, 1,024 (23%) tested positive on CLART; 822 (19%) on HC2; and 241 (5%) had ≥ ASCUS (Table 4). Among the 23-29 year olds, 7% had ≥ ASCUS, and among 30-65 year olds that was the case in a relatively stable proportion of 4% (Figure 2). On the other hand, the proportion of women with a positive CLART test and normal cytology was strongly age-dependent, and it decreased from 31% (400/1,287) at age 23-29 years, to 18% (228/1,299) at 30-39 years, 11% (96/904) at 40-49 years, 8% (37/465) at age 50-59 years, and 8% (17/215) at age 60-65 years (P < 0.0001).

**Table 4:** **CLART results for 4,410 primary** ^**a**^ **samples, by age, screening history, cytology, and HC2 results**

	Outcomes on CLART, N (%)				
	Any high-risk genotype (%)^b^	No high-risk genotypes (%)^c^	Only low-risk genotypes (%)^b^	Invalid (%)	Total (%)
**Total**	1,024 (23.2%)	3,375 (76.5%)	528 (12.0%)	11 (0.2%)	4,410 (100%)
**Age (years)**					
16-22	77 (57.0%)	58 (43.0%)	18 (13.3%)	0 (0.0%)	135 (100%)
23-29	485 (37.7%)	801 (62.2%)	175 (13.6%)	1 (0.1%)	1,287 (100%)
30-39	274 (21.1%)	1,021 (78.6%)	139 (10.7%)	4 (0.3%)	1,299 (100%)
40-49	116 (12.8%)	783 (86.6%)	104 (11.5%)	5 (0.6%)	904 (100%)
50-59	45 (9.7%)	419 (90.1%)	55 (11.8%)	1 (0.2%)	465 (100%)
60-65	19 (8.8%)	196 (91.2%)	22 (10.2%)	0 (0.0%)	215 (100%)
>65	8 (7.6%)	97 (92.4%)	15 (14.3%)	0 (0.0%)	105 (100%)
**Cytology**					
Normal	847 (20.4%)	3,290 (79.3%)	494 (11.9%)	11 (0.3%)	4,148 (100%)
ASCUS	49 (57.6%)	36 (42.4%)	9 (10.6%)	0 (0.0%)	85 (100%)
LSIL	64 (73.6%)	23 (26.4%)	20 (23.0%)	0 (0.0%)	87 (100%)
≥HSIL	64 (92.8%)	5 (7.2%)	2 (2.9%)	0 (0.0%)	69 (100%)
≥ASCUS	177 (73.4%)	64 (26.6%)	31 (12.9%)	0 (0.0%)	241 (100%)
Inadequate	0 (0.0%)	21 (100%)	3 (14.3%)	0 (0.0%)	21 (100%)
**HC2** ^**d**^					
Positive	693 (84.3%)	128 (15.6%)	84 (10.2%)	1 (0.1%)	822 (100%)
Negative	330 (9.2%)	3,245 (90.5%)	444 (12.4%)	10 (0.3%)	3,585 (100%)

*Abbreviations:* *ASCUS* atypical squamous cells of undetermined significance, *HC2* Hybrid Capture 2 assay, *HPV* Human Papillomavirus, *≥HSIL* high-grade intraepithelial lesions or worse, *LSIL* low-grade squamous intraepithelial lesions.

^a^Predominantly screening samples, including a small proportion of samples taken for indication.

^b^According to classification reported by IARC [2].

^c^Considered negative on the CLART assay.

^d^Three samples were not tested with HC2.

### Intra-laboratory reproducibility of CLART

Out of 273 samples collected for evaluation of reproducibility, showing no high-risk or low-risk genotypes in the first run, 256 (94%) could be reproduced. Among the 17 (6%) non-reproducible samples, 8 showed high-risk infections in the second run. Hence, when only high-risk infections were considered as a positive result in the second run, the negative reproducibility was 97% (264/273).

Among the 371 samples in which at least one of the 35 HPV genotypes was detected by CLART in the first run, 329 (89%) still had at least one genotype detectable in the second run. No genotype was detected in the second run among the remaining 42 (11%) samples, of which 28 initially showed infections with at least one high-risk genotype. Restricting the evaluation to the 283 samples with high-risk genotypes detected in the first run, 243 (86%) reproduced a high-risk genotype. An HPV 16 result from the first run (n = 71) was reproduced in 67 (94%) samples, and for HPV 18 (n = 30) this was the case in 25 (83%) samples.

Assuming that in routine work, 25% had high-risk HPV on CLART (Table 2), 94% (= (25% × 86%) + (75% × 97%)) of all samples would have a reproducible result. For women with screening samples at age 30-65 years, 16% tested positive on CLART (Table 3), and 95% (= (16% × 86%) + (84% × 97%)) would be reproducible.

## Discussion

The Horizon study is the first population-based study to quantify cervical HPV infections using CLART HPV2 genotyping assay with cross comparison to HC2 on unselected screening samples stored in SurePath. Denmark has a high-risk, but well screened, population with respect to cervical cancer, and CLART detected at least one high-risk genotype in 25% of the samples, compared to 20% by HC2. The proportion testing positive decreased strongly with women’s age. In primary screening of women aged ≥30 years, 16% tested positive on CLART, and 12% on HC2; and most of these women had normal cytology. HPV 16 causing more than half of cervical cancers [22], was found in 7% of women. Additionally, 12% of women were infected with only low-risk genotypes.

We also evaluated CLART’s laboratory performance. Here, focus was on intra-laboratory reproducibility, as a lack thereof may pose problems for laboratories, e.g. in terms of their ability to verify the initial findings. In total, 94% of samples initially negative on all 35 genotypes were negative at retesting. When only high-risk genotypes were considered, this was 97%. Similarly, 89% of samples initially testing positive for at least one of the 35 genotypes, were positive at retesting. When only high-risk genotypes were considered, this was 86%. Taking the proportions of HPV positive and negative samples into account, overall 94% of samples were reproducible. This was similar to the reproducibility evaluated in the Horizon study of two other newly available commercial assays, Roche’s cobas [19] and Hologic/Gen-Probe’s APTIMA [18]. In a small Portuguese study of CLART (n = 75), 99% of archived ThinPrep samples were reproducible [12]. The proportion of positive samples was not reported from that study. We conclude that by using an approach including a pre-warming step to counter any cross linking effect on a sample’s genomic material by SurePath, CLART was reproducible. This protocol modification was agreed upon with the manufacturer prior to the study. The differences to previous findings on ThinPrep appear relatively small, though SurePath due to its formaldehyde content poses a larger challenge to molecular HPV assays than the methanol based ThinPrep.

We had to exclude 19% of the samples owing to insufficient volume of available residual material. However, there was no significant difference between the included and the excluded samples in terms of the women’s age, cytology, and the HC2 outcome. All samples were tested in one laboratory by the same staff, and the instrumentation was used as supplied and maintained by the manufacturers. Although there may be some differences between automated and manual DNA extraction, we used the former method in our study. This allowed us to evaluate CLART in a relatively high throughput fashion. This is important as manual extraction in a laboratory routinely running >10,000 HPV tests per year would be feasible neither from a patient safety nor from a quality control/quality assurance point of view. Although SurePath is a widely used screening medium for sample collection, no previous study has addressed the use of CLART technology for HPV detection on this media type, using automated extraction and testing.

In 2004-2005, a total of 11,617 consecutive (screening) SurePath samples evaluated in the same laboratory were tested with HC2 [15]. The median age of the women in that study was 36.4 years (range: 15-93), and 6% had abnormal cytology. The proportion of women aged 25-64 years testing positive on HC2 was ~17%, which is similar to 16% in Horizon. Horizon results are therefore in good agreement with the earlier data from the same population.

So far CLART’s clinical performance has only been evaluated in a handful of studies, and several of these did not compare with that of HC2 [8, 13, 23–26]. In 405 Portuguese women aged 25-63 years attending colposcopy for cervical abnormalities, 268 (63%) tested positive on CLART (13 high-risk genotypes), and 274 (64%) tested positive on HC2. In total, 99% of the samples were concordant between CLART and HC2. The sensitivity for ≥ CIN2 of both assays was 96% (95% CI: 93-98), and the specificities were comparable, 74% (95% CI: 67-80) for CLART, and 71% (95% CI: 64-78) for HC2 [12]. In a UK study including 953 women referred for colposcopy after cytological abnormalities, the sensitivity of CLART for ≥ CIN3, 84% (95% CI: 77-89), was lower than that of HC2, 99% (95% CI: 97-100). CLART’s specificity was higher than that of HC2, 36% (95% CI: 32-40) vs. 25% (95% CI: 22-29), respectively [9]. The authors though mentioned technical problems in evaluation of CLART, and suggested caution in interpreting these results. In the Horizon study, the histological follow-up of women with positive screening tests is underway. Following routine recommendations in Denmark, women with abnormal cytology were offered additional testing. In addition, we invited women with normal cytology and at least one positive HPV test for repeated testing after 18 months. Standard measures of sensitivity and specificity will be reported once follow-up data are available.

Experts largely discourage use of HPV DNA testing in routine screening of women below age 30 years [27]. In our study, 38% of screening samples from women aged 23-29 years tested positive on CLART, and 33% on HC2. In comparison, HPV DNA testing in women aged 30-65 years showed that 16% of screened women tested positive on CLART, 12% on HC2, and 4.4% on LBC (Figure 2). Recent data for Copenhagen, retrieved from the national Pathology Data Bank, showed that 1.3% of screened women had ≥ CIN3 detected following abnormal cytology. If HPV testing is 32% more sensitive for ≥ CIN3 than cytology [28], about 1.7% of women are expected to have ≥ CIN3 detected after a positive HPV test. Thus, 14.0% (=15.7-1.7) of women aged 30-65 years are expected to get a false-positive CLART test, defined as a positive CLART test without a subsequent diagnosis of ≥ CIN3. On HC2, 10.0% (=11.7-1.7) had a false-positive test, and 3.1% (=4.4-1.3) on LBC. The lifetime background risk of cervical cancer in Danish women is estimated at <4% [29]. Hence, the very high proportions of women with false-positive HPV tests, on CLART and HC2 alike, per screening round represent a challenge for the implementation of primary HPV screening in women above the age of 30 years.

Several marketed HPV assays have partial or full genotyping. The added value for screening of detailed genotyping is widely debated, yet it has been suggested to use genotyping in triage of HPV-positive women to colposcopy [30]. However, from a screening perspective, the main advantage of HPV genotyping would be to reduce the number of false-positive tests sometimes seen with cross-reactivity between high-risk probes/primers and low-risk genotypes. In our study, this was exemplified by 160 samples (3% of all samples) positive on HC2 but negative on high-risk CLART genotypes, of which 111 (2% of all samples) tested positive on low-risk CLART genotypes. Moreover, HPV 66, a genotype with limited evidence for carcinogenicity (group 2B carcinogen) [2], and detectable by e.g. cobas, Abbott RealTi*m*e HPV test, BD Onclarity and APTIMA, was found in 3% (157/5,068; Table 1) of our samples; and in about half of these samples (72/157), no high-risk genotype was detected. This raises a question on whether inclusion of HPV 66 in non-genotyping assays may result in unnecessary follow-up and referrals for colposcopy. In 8% of all samples, CLART detected high-risk genotypes whereas HC2 returned a negative result (Table 2), probably due to the difference in HPV detection technology, CLART being a PCR-based assay and HC2 a hybridization assay. The significance of these discordant tests will be explored when follow-up data are available.

In monitoring the effect of HPV vaccination, the use of genotyping assays will be very important. HPV vaccination is expected to change the dynamics of the screening programs by decreasing the background incidence of cervical cancer and hence also the need for treatment of CIN [31]. Until now, however, only a few countries have implemented organised monitoring of vaccine impact. Using genotyping assays, the task of screening and vaccine monitoring could be combined, lowering the overall costs. For this to become a reality, however, it will be necessary to firmly establish that the genotyping assays perform equally well on both sensitivity and specificity as the currently widely used non-genotyping assays like HC2.

## Conclusions

In summary, this is the first investigation of performance of the CLART HPV2 genotyping assay in a population-based study using unselected screening samples collected in SurePath. CLART performed well with positive and negative reproducibility comparable to that of other commercially available assays. Moreover, CLART showed a higher positivity rate for 13 high-risk HPV genotypes than HC2. As HPV-vaccinated birth cohorts are soon to enter screening age, our study maps the last pre-vaccination HPV genotype distribution in Danish women, and can as such serve as reference for the monitoring vaccine impact.

## Authors’ original submitted files for images

Below are the links to the authors’ original submitted files for images. Authors’ original file for figure 1 Authors’ original file for figure 2
