# Supplementary figures and images for: HPV prevalence and genotype distribution in a population-based split-sample study of well-screened women using CLART HPV2 Human Papillomavirus genotype microarray system
Source: BMC Infect Dis. 2014 Jul 26;14:413. doi: 10.1186/1471-2334-14-413 (PMC4122758; doi:10.1186/1471-2334-14-413)

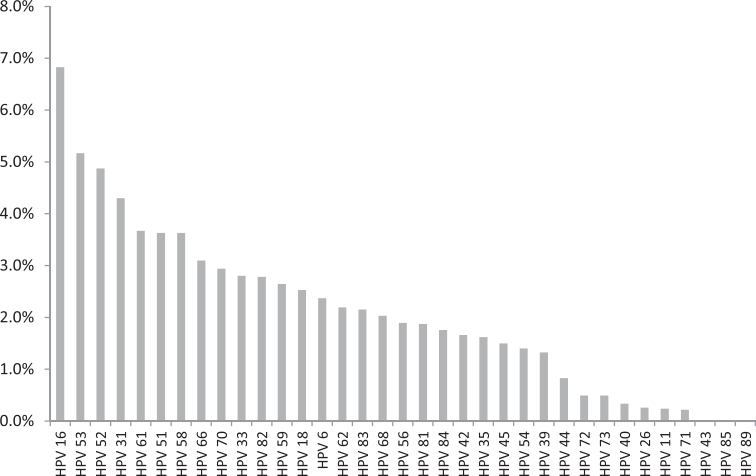

Supplement: Supplementary file 1 — Authors’ original file for figure 1 [file 12879_2013_3714_MOESM1_ESM.pdf]

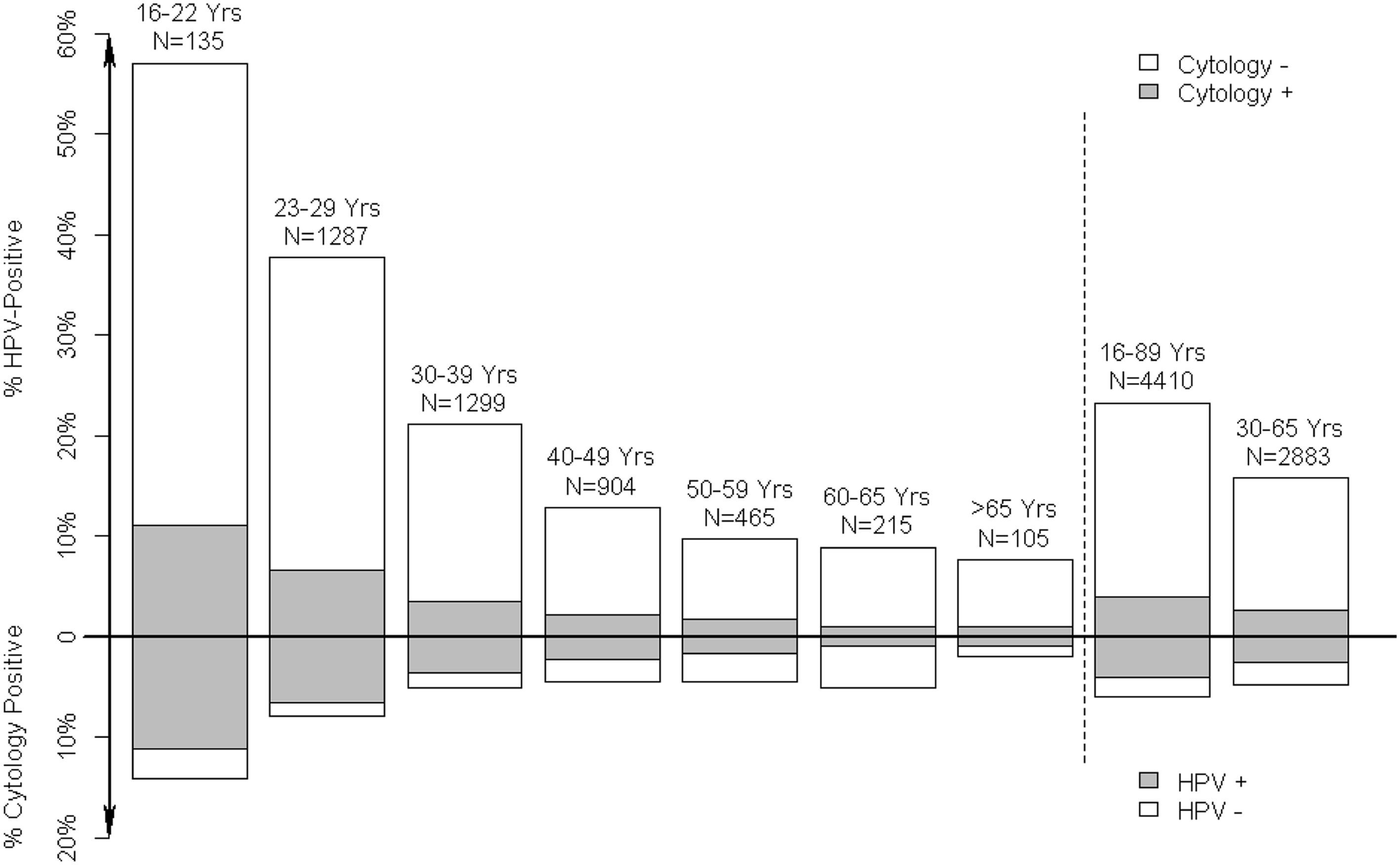

Supplement: Supplementary file 2 — Authors’ original file for figure 2 [file 12879_2013_3714_MOESM2_ESM.tif]
